# “An Eye for an Eye”? Neural Correlates of Retribution and Forgiveness

**DOI:** 10.1371/journal.pone.0073519

**Published:** 2013-08-29

**Authors:** Martin Brüne, Georg Juckel, Björn Enzi

**Affiliations:** 1 Department of Psychiatry, Psychotherapy and Preventive Medicine, Landschaftsverband Westfalen Lippe University Hospital, Ruhr-University, Bochum, Germany; 2 Division of Cognitive Neuropsychiatry and Psychiatric Preventive Medicine, Department of Psychiatry, Psychotherapy and Preventive Medicine, Landschaftsverband Westfalen Lippe University Hospital, Ruhr-University, Bochum, Germany; University of Groningen, Netherlands

## Abstract

Humans have evolved strong preferences for equity and fairness. Neuroimaging studies suggest that punishing unfairness is associated with the activation of a neural network comprising the anterior cingulate cortex, anterior insula, the ventral striatum, and the dorsolateral prefrontal cortex (DLPFC). Here, we report the neuronal correlates of retribution and “forgiveness” in a scenario, in which individuals first acted as a recipient in an Ultimatum Game, and subsequently assumed the position of a proposer in a Dictator Game played against the same opponents as in the Ultimatum Game. Most subjects responded in a tit-for-tat fashion, which was accompanied by activation of the ventral striatum, corroborating previous findings that punishing unfair behaviour has a rewarding connotation. Subjects distinguished between the human opponent and computer condition by activation of the ventromedial PFC in the human condition, indicative of mentalising. A substantial number of subjects did not retaliate. Neurally, this “forgiveness” behaviour was associated with the activation of the right (and to a lesser degree left) DLPFC, a region that serves as a cognitive control region and thus may be involved in inhibiting emotional responses against unfairness.

## Introduction

Research suggests that humans have evolved preferences for equity and fairness, which has strongly impacted the emergence of complex moral emotions such as shame, guilt, trust, gratitude and moralistic aggression [1,2]. In fact, mutual cooperation and the implementation of rules of exchange is characteristic of all human societies and has left its mark on judicial systems across cultures [3], as reflected in the oldest written testimonials such as the Babylonian code of Hammurabi [4]. According to these inscriptions and documented evidence from Christian, Jewish and Islamic law, violation of social norms was punished following a relatively simple “tit-for-tat”-like rule (“an eye for an eye…”). Expressed in the language of game theory [5], while cooperation and reciprocity are highly valued, at least among in-group members, defection is usually shunned or punished. Conversely, most religions have also implemented rules when to forgive a transgression of social rules and moral values. However, forgiveness is usually only granted if preceded by repentance of the person who disobeyed to the social rules, but not for “free”. In any event, both retribution and forgiveness may have complementary biological functions of preventing defectors from future harm-doing or withholding benefits in the future [6].

Interpersonal scenarios involving reciprocality, trust, or fairness between two parties have experimentally been modelled in a variety of economic games. Studies utilising the prisoners’ dilemma (PD) game, for example, have demonstrated that it seems to pay-off to be “nice”, at least when the game is played iteratively [7], which in theory is not the optimal strategy, because without knowing the strategy chosen by the other player, non-cooperation is associated with a higher expectancy value. Interestingly, most people nevertheless cooperate in the PD at a considerable rate [8]. These findings suggest that evolutionary stability of cooperation necessitates the ability to identify individuals who do not cooperate (referred to as “defectors” or “cheaters”) [9], if cooperation is to be maintained among genetically unrelated individuals. In addition, to reinstall cooperation after a violation of rules of exchange has occurred, it is required that effective punishment can be imposed on those who intentionally deviated from social norms [1,10].

Functional brain imaging studies into neuroeconomics have revealed that humans possess a neural network that is engaged in the evaluation of violations of cooperative and reciprocal principles (reviewed in 8,11). In one of the first studies, Sanfey et al. [12] discovered that individuals, who acted as recipients in an Ultimatum Game (UG), activated the anterior insula (AI) and the anterior cingulate cortex (ACC) more strongly when receiving unfair offers, as compared to fair offers. In a UG, one player (the proposer; “A”) is asked to propose how to distribute an amount of money, while player “B” (the recipient) has the option to either accept or decline the offer. If B agrees, the sum will be split according to A’ s proposal. If, B rejects, however, both receive nothing [13]. Rejecting unfair offers can therefore be seen as a mild form of social punishment [14,15,16]. Interestingly, Sanfey et al. [12] found that the activation of the dorsolateral prefrontal cortex (DLPFC) was greater relative to insula activation, when unfair offers were accepted, whereas the reverse relationship was observed when unfair offers were subsequently rejected. In a similar vein, Tabibnia et al. [17] reported an increased activation of the ventrolateral prefrontal cortex and a decreased insular activation when unfair offers were accepted. Notably, receiving fair offers led to an activation of the ventral striatum, a region that is involved in reward processing [17]. This is consistent with the interpretation that regions in the prefrontal cortex are involved in controlling emotional responses to perceived unfairness [12] possibly by down-regulating the insula [17], which is thought to be a key structure in interoceptive awareness and the representation of negative emotions such as anger, disgust, as well as guilt, shame and other “moral” emotions [18]. Alternatively, it has been suggested on the basis of experiments using repetitive transcranial magnectic stimulation (rTMS) that the DLPFC is more directly involved in implementing culturally acquired fairness norms and in suppressing selfish tendencies, because functional inhibition of the right DLPFC has been shown to increase acceptance rates in a UG [19].

With regard to the activation pattern during punishment of unfair behaviour, neuroimaging research has shown that taking corrective action in the form of retaliation or social punishment is associated with activation of the ventral striatum, a region of the brain that is involved in encoding reward anticipation [20], especially, when punishment is effective and not just symbolic [10]. Put another way, revenge literally tastes “sweet” [21], and is often associated with feelings of pleasure. The activation of the reward system during moralistic aggression suggests that the evolved function of retribution probably resides in the fact that it deters cheaters from further non-cooperation, and hence, forces the defector back into a reciprocating relationship [6]. If this were the case, one could argue that people would be expected to spontaneously respond to perceived unfairness by not reciprocating. However, since retribution can be costly to the punisher (e.g., by turning a friend into a foe) people may be willing to refrain from punishing experienced unfairness and instead, take reparative action [6]. Assumed that there is adaptive value of “forgiveness” (by reducing the cost of revenge), it would be plausible to expect that reconciliatory action were accompanied by an activation of the reward system. Indirect support for this comes from studies showing that striatal activity can be observed when making charitable donations, especially if made voluntarily, as compared to a tax imposed by an authority [22,23,24]. Alternatively, the decision not to punish, rather than being rewarding in itself, may require cognitive effort to inhibit retaliatory action, which would be associated with an activation of the DLPFC, but not necessarily with striatal activity.

Accordingly, we designed a study in which subjects initially played a UG in the role of the recipient against four players, two of which made fair offers, and the other two unfair offers. In the second part of the experiment, the roles were reversed such that participants – now in the role of the proposer in a Dictator Game (DG) – had the choice to act in fair or unfair ways towards the previously fair or unfair proposers in the UG. A DG was chosen for the second round, because individuals usually offer less in a DG (around 28 percent) than in an UG (around 40 percent), such that fair behaviour in a DG can be considered more altruistic than making fair offers in a UG, and hence, better reflects one’s willingness not to punish [25]. We predicted, based on previous studies, that participants would show a pattern of activation involving the insula, the ventral striatum, and the DLPFC when playing a UG. We further hypothesised that retribution would be accompanied by activation of the ventral striatum, as a biological substrate of reward from retaliation. Finally, we expected that a smaller number of subjects would refrain from retaliation, and instead, treat previously unfair opponents fairly, which would be associated with activation of the DLPFC, and possibly with striatal activation.

## Material and Methods

### Ethics statement

The study was approved by the Ethics Committee of the Medical Faculty of the Ruhr-University, Bochum. All participants gave written informed consent. The investigation was conducted in full accordance with the principles expressed in the Declaration of Helsinki.

### Subjects

Twenty-nine healthy subjects with no psychiatric, neurological or medical illnesses (16 male and 13 female subjects, average age 27.9 ± 4.5 years, range 21 to 37 years, all right-handed) were enrolled in the study.

### Behavioural and neuropsychological data

The behavioural data (reaction times, number of trials, acceptance and punishment rates) were extracted using perl (www.perl.org). Further analyses regarding behavioural data (t-test for paired samples) were carried out using SPSS 11 (SPSS Inc., Chicago, USA).

### Experimental paradigm

**Figure 1:**
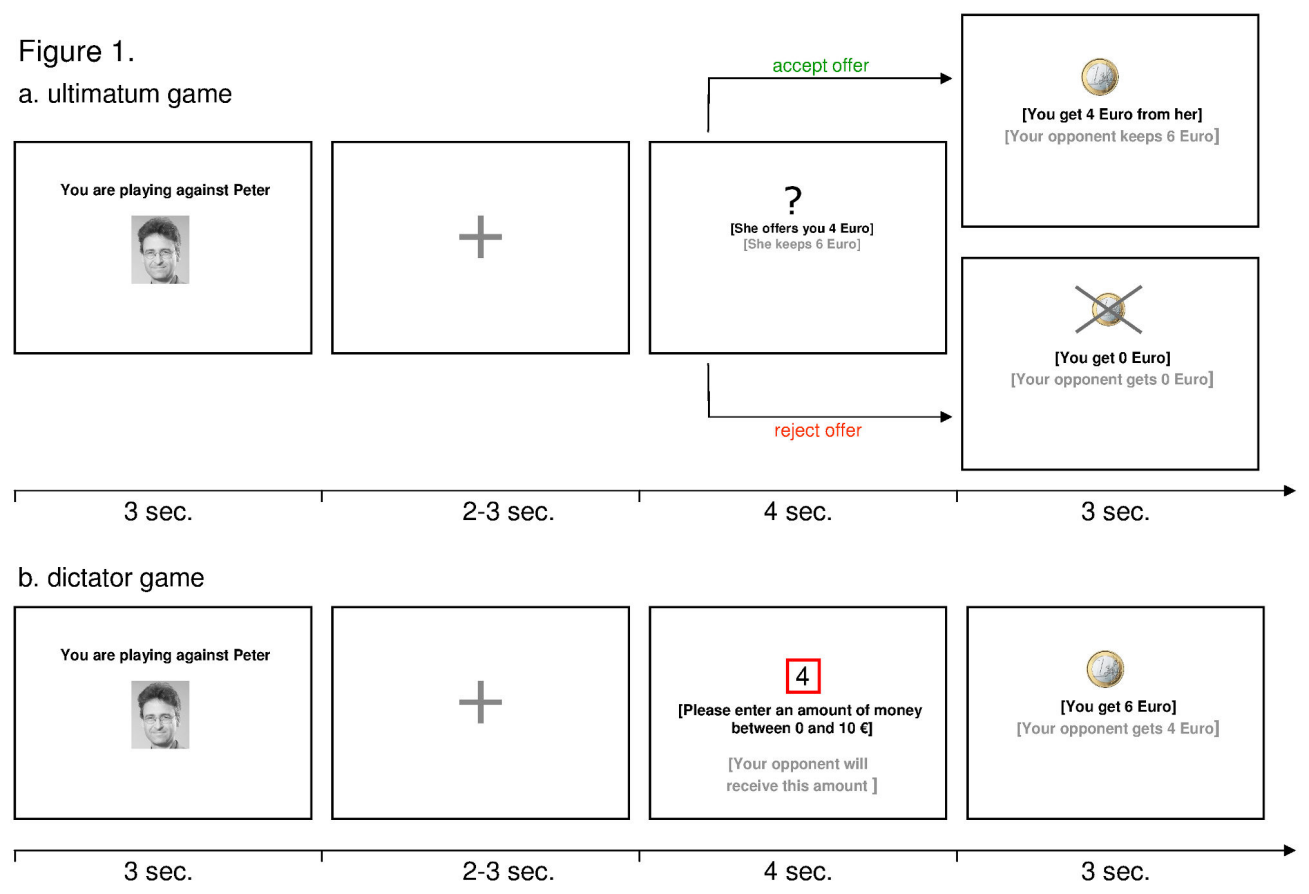
Design and schematic structure of the fMRI paradigm (The subject of the photograph has given written informed consent, as outlined in the PLOS consent form, to publication of his photograph). a. Ultimatum game: Subjects played against six different opponents (two male human players, two female human players and two computer players), who offered an amount of money varying between zero € and five €. Nevertheless, subjects had the possibility to accept or to reject the offered amount of money by pressing a button. Finally, the subjects were informed by a visual feedback about the accepted or rejected amount of money. b. Dictator game: In the second run of the experiment, subjects played against the previously introduced six opponents. In contrast to the ultimatum game presented in the first run, subjects had now the possibility to share the money according to their needs and beliefs. For this reason, our subjects were in the position to punish or to reward previously unfair or fair opponents. Finally, subjects were informed by a visual feedback about their gain in this trial and the amount of money assigned to the opponent. All trials were separated by a 4.0-5.0s intertrial interval (ITI). In addition, eight separate baseline events varying between 4.0 and 5.5 seconds were presented per run.

The applied functional imaging paradigm consists of two different tasks. Each run consisted of 90 experimental trials (30 ‘fair human opponent’ trials, 30 ‘unfair human opponent’ trials, and 30 ‘computer opponent’ trials) with a total duration of approximately 27 minutes. In the first run, all subjects completed a modified version of an Ultimatum Game [12,26]. In the UG, subjects assumed the role of the responder against four different virtual human characters (two male, two female) and a computer opponent (control condition). Participants were instructed that such scenarios have been applied “online” in similar studies, but that the games were played offline. Debriefing after the experiment revealed that subjects had little difficulties in appreciating that the scenarios depicted interpersonal “real-life” behaviours, and that they were well aware of the difference between the human and the computer conditions. The offers made by the proposers were splits of ten €. First, the subjects saw the picture of their opponent (“viewing period” with a duration of 3 seconds), followed by a jittered 2-3 seconds anticipation period. In the following decision period, the offer proposed by the opponent was presented for 4 seconds. Within this timeframe subjects could accept or reject the offered amount of money via button press. If the offered money was rejected, both players received zero €. Finally, the subjects were informed by a visual 3-second-feedback about the accepted or rejected amount of money. All trials were separated by a 4.0-5.0 seconds intertrial interval. In addition, eight separate baseline events (no decision-making involved) varying between 4.0 and 5.5 seconds were presented (Figure 1a). In the UG, the human characters either made consistently (relatively) fair offers or unfair offers, i.e. the two fair opponents (one male, one female) offered splits of 5:5 €, 4:6 €, and 3:7 €, whereas the two unfair opponents (one male, one female) offered splits of 2:8 €, 1:9 €, and 0: ten €. The subjects were left unbeknownst to the individual strategy of the proposers in the UG, that is, it was the participants’ implicit task to recognise whether a proposer was fair or unfair. In contrast to the behaviour shown by the human opponents, the computer player offered money splits generated by chance, i.e. offers covered the full range between 5:5 € and 0: ten € splits.

In the second game, which was designed as a Dictator Game (DG), all subjects played against the previously introduced (human and computer) opponents in the role of the proposer. The trial structure, and thus the duration of all relevant events, was comparable to the UG. After a 3-second viewing period and a 2-3-second anticipation period, subjects were told to assign money varying between zero € and ten € to their opponents within a 4-second decision period. For this purpose an MRI-compatible button-box was used. Finally, subjects were informed by a visual feedback about their gain in this trial and the amount of money assigned to the opponent. As in the UG, all trials were separated by a jittered 4-5s intertrial interval, additionally eight baseline events of 4-5 seconds duration were presented randomly (Figure 1).

Subjects were instructed that all human opponents represented real human characters and that the subjects’ decision in the UG and the DG would influence the monetary gain of their opponents, as well as their own payment. The order of offers was generated randomly by the computer and all subjects received a fixed compensation of twenty-five €. In the DG, subjects were able to share the money according to their feelings, and thus to punish or to reward previously unfair or fair opponents.

The experiment was presented via MRI-compatible LCD-goggles (Resonance Technology Inc., Los Angeles, CA, USA) using the “presentation” software package (Neurobehavioral Systems Inc., Albany, CA, USA).

### fMRI data acquisition and analysis

Functional data was collected using a 3-Tesla whole body MRI system (Philips Achieva 3.0T TX) equipped with a 32-channel Philips SENSE head coil. 32 T2*-weighted echo-planar (EPI) images per volume with blood-oxygen level dependent (BOLD) contrast were obtained using a sensitivity encoded single-shot echo-planar imaging protocol (SENSE-sshEPI; matrix 80 x 80 mm^2^, reconstructed to 112 x 112 mm^2^, field-of-view 220 x 220 mm^2^, in-plane resolution 2.75 x 2.75 mm^2^, slice thickness 3 mm with 1 mm gap, reconstructed to a final voxel size of 1.96 x 1.96 x 3 mm^3^, TR = 3000 ms, TE = 35 ms, flip angle α = 90°, SENSE factor R_AP_ = 2.0). The slices were acquired in interleaved order parallel to the bi-commissural plane and provided whole-brain coverage. Subjects had to complete two scanning runs with 530 volumes per run. The first five volumes were discarded due to saturation effects. Prior to the functional scanning session, a high-resolution, T1-weighted anatomical gradient echo scan was acquired for each subject (3D-TFE; matrix 300 x 235 mm^2^, reconstructed to 320 x 320 mm^2^, field-of-view 240 x 188.8 x 192 mm^3^, in-plane resolution 0.8 x 0.8 mm^2^, slice thickness 0.8 mm, reconstructed to a final voxel size of 0.75 x 0.75 x 0.8 mm^3^. In total, 240 slices in transverse orientation were acquired. TR = 10 ms, TE = 4.6 ms, flip angle α = 8°, SENSE factor R_RL_ = 2.5 and R_FH_ = 2.0).

The functional data were preprocessed and statistically analyzed using the SPM8 software package (Wellcome Department of Cognitive Neuroscience, University College London, UK; http://www.fil.ion.ucl.ac.uk) and MATLAB 7.11 (The Mathworks Inc, Natick, MA, USA). After temporal correction and correction for between-scan motion artefacts by realignment to the first volume, the anatomical scan was co-registered to a mean functional image. The normalization was generated by warping the subject’s anatomical T1-weighted scan on the T1-template provided by SPM8 (MNI stereotactic space) and applying these parameters to all functional images. The images were re-sampled to a final voxel size of 3 x 3 x 3 mm^3^ and smoothed with an isotropic 10 mm full-width half-maximum Gaussian kernel. The time-series fMRI data were filtered to eliminate low-frequency signal drifts using a high pass filter (cut-off 100 seconds).

In line with previous research [12,26], we focused our analysis on the decision period of the UG and DG. For the UG, the decision period for fair and unfair human opponents, as well as for computer opponents, was modelled, resulting in four experimental conditions or regressors (fair human offer, unfair human offer, fair computer offer, unfair computer offer). In addition, the six realignment parameters were included as regressors of no interest. As regards the DG, the decision period was categorized according to the subject’s behaviour, i.e. previously fair human opponent treated fairly, previously unfair human opponent treated unfairly, computer opponent treated fairly, computer opponent treated unfairly. These four conditions were entered as regressors in our design matrix. Additionally, the six realignment parameters were entered as regressors of no interest, resulting in 10 conditions. A statistical model for each subject was computed by convolving a canonical haemodynamic response function with the above-mentioned design [27]. All further statistical analyses followed the general linear model approach [28]. Regionally specific condition effects were tested by employing linear contrasts for each subject and each condition of interest, followed by a second level random effects analysis using the full-factorial option implemented in SPM8 (factors ‘opponent’ [human, computer] and ‘behaviour’ [fair, unfair]).

The contrast [UG: main effect of human opponent fair + unfair] was calculated for validation of the obtained results. These results were displayed using the MRIcron software package (http://www.mccauslandcenter.sc.edu/mricro/mricron). For delineation of regional responses associated with fairness and unfairness, the contrast ‘UG: positive interaction opponent x performance’ was calculated. The resulting statistical parametric maps were thresholded at p [uncorrected] < 0.001 for k > 10 voxel. We subsequently concentrated all further analyses on five regions of interest (ROI), i.e. the bilateral ventral striatum, the bilateral ventromedial prefrontal cortex, and the right insula. Only activations surviving a family-wise error correction (p[FWE] < 0.05) after Small Volume Correction were reported.

In this context, the decision period of the UG was used as functional localiser [29] to ensure the valid and independent statistical analysis of the DG decision period [30]. Using sphere shaped regions of interest (ROI; radius 5 mm) centred upon the peak voxel within each area of interest, signal changes (contrast estimates in arbitrary units [a.u.]) derived from the decision period of the DG (fair human opponent treated fairly, unfair human opponent treated unfairly, computer opponent treated fairly, computer opponent treated unfairly) were extracted using the “rfxplot”-toolbox (http://rfxplot.sourceforge.net/) for SPM [31].

Since 12 subjects showed a tendency to treat previously unfair opponents in a fair way, a second design matrix was calculated for these subjects including the above-mentioned regressors as well as the condition ‘unfair human opponent treated fairly’. Contrast specification and signal change extraction was carried out as mentioned above. It should be noted that we concentrated this analysis on the bilateral dorsolateral prefrontal cortex, which are involved in controlling prepotent emotional responses to observed unfairness. For this purpose, the 2nd-level contrast ‘UG: positive effect of all conditions’ (p[FWE] < 0.05, k > 20) was used for signal change extraction.

Regarding the DG, two subjects were excluded from the ROI-analysis due to excessive head movement in the second scanning run, and another three subjects were excluded for technical reasons (defect of the MRI-compatible LCD-goggles in the second scanning run). For anatomical localization an averaged structural scan of all subjects was calculated. All further statistical analyses (t-tests for dependent samples) were calculated using the software package SPSS 11.5 (SPSS Inc., Chicago, USA). Data storage and availability is guaranteed according to the recommendations of the German Research Foundation (DFG).

## Results

### Behavioural data

As expected, we observed a decline in the acceptance rate according to the fairness of the offer (5:5€-offers were accepted in 97.4% (± 4.7%), 4:6€-offer in 86.0% (± 25.9%), 3:7€-offer in 60.7% (± 36.6%), 2:8€-offer in 24.2% (± 32.4%), 1:9€-offer in 15.1% (± 26.2%), and 0: ten €-offers were accepted in 4.00% (± 10.6%), indicating that the paradigm produced comparable results to previous studies. The acceptance rates were calculated in relation to the total number of trials. Acceptance rates differed significantly between more fair (5:5€, 4:6€, and 3:7€) and more unfair (2:8€, 1:9€, and 0: ten €) conditions in both groups, i.e. playing the UG against human opponents or against the computer (human: t_28_ = 18.055; p < 0.001; computer: t_28_ = 17.569; p < 0.001).

**Figure 2:**
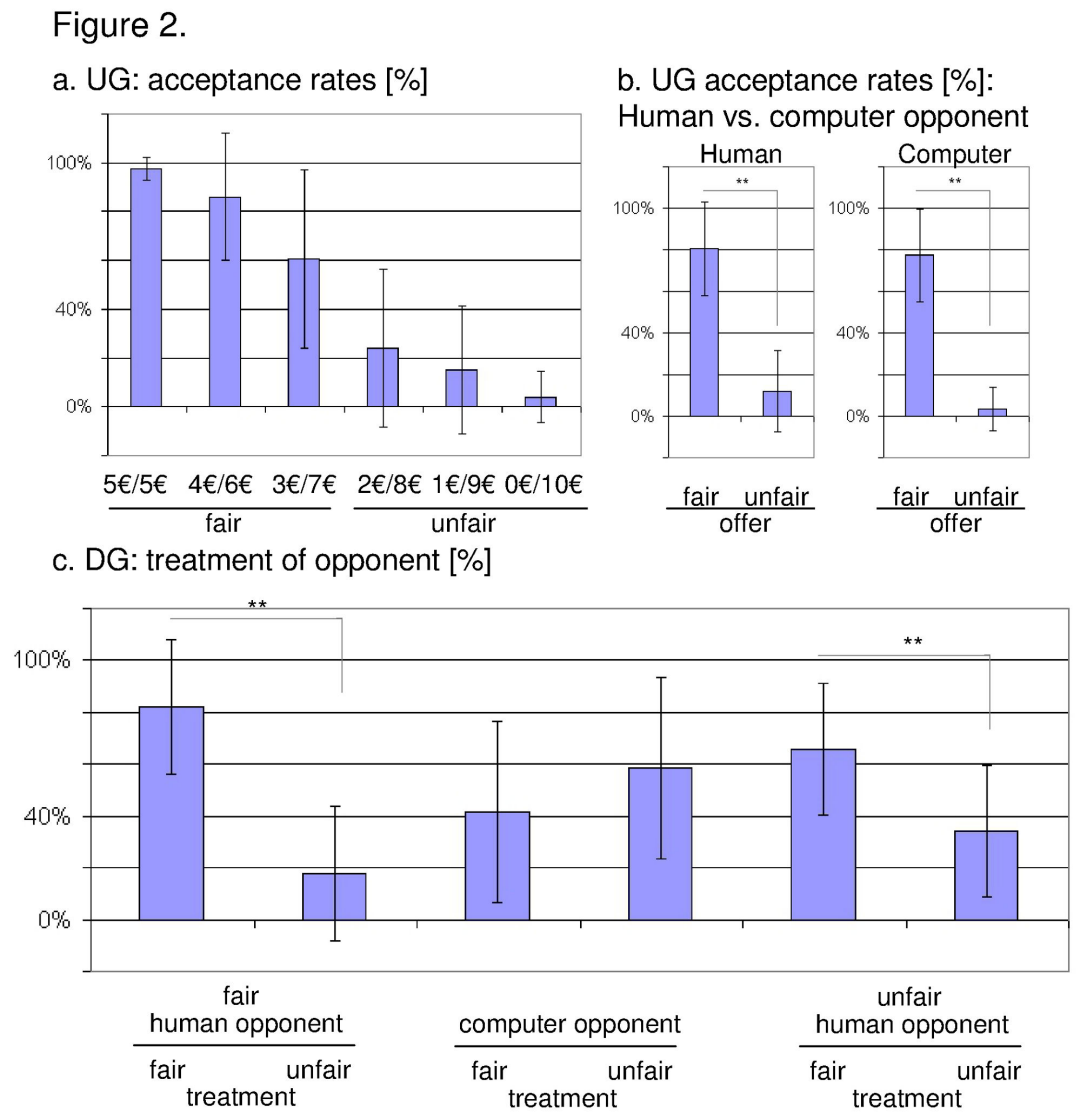
Behavioural data derived from the (a) ultimatum game and from the (b) dictator game. a. Acceptance rates in percent categorized according the amount of money offered to the subject. Furthermore, acceptance rates for fair offers, i.e. five €-, four €, and three €-trials, and unfair offers, i.e. two €-, one €-, and zero €-trials, were presented on the right side of the diagram. b. Treatment of opponents in the dictator game. Concerning the dictator game, we calculated the ratio (expressed in percent) between the number of trials representing a fair treatment of a previously fair player, unfair treatment of a fair player, fair treatment of a computer player, unfair treatment of a computer player, unfair treatment of an unfair player, and fair treatment of an unfair player, respectively, and the total number of trials presented in this part of the experimental paradigm. ** p < 0.01; * p < 0.05. Error bar represents standard deviation.

With regard to the DG, previously fair human opponents were treated fairly in 82.2%, whereas 17.8% were treated unfairly. As regards unfair human opponents, 65.9% were treated unfairly, as opposed to 34.1% of previously unfair proposers treated fairly (Figure 2c). In addition, we calculated a 2-by-3 repeated measures ANOVA with the factors 'treatment' (fair, unfair) and 'opponent' (categorized according to the behaviour in the UG, i.e. previously fair, previously unfair, computer). Significant results emerged regarding the interaction 'treatment x opponent' (F_2,46_ = 22.731; p < 0.001) and the main effect of 'opponent' (F_2,46_ = 3.797; p = 0.03), whereas the main effect of 'treatment' was not significant. A more detailed analysis revealed that previously fair players were treated more often fairly (t_23_ = 6.159, p < 0.001), in addition previously unfair opponents received significantly more punishment (t_23_ = 3.055, p = 0.006) (Figure 2c).

### Functional imaging data

**Figure 3:**
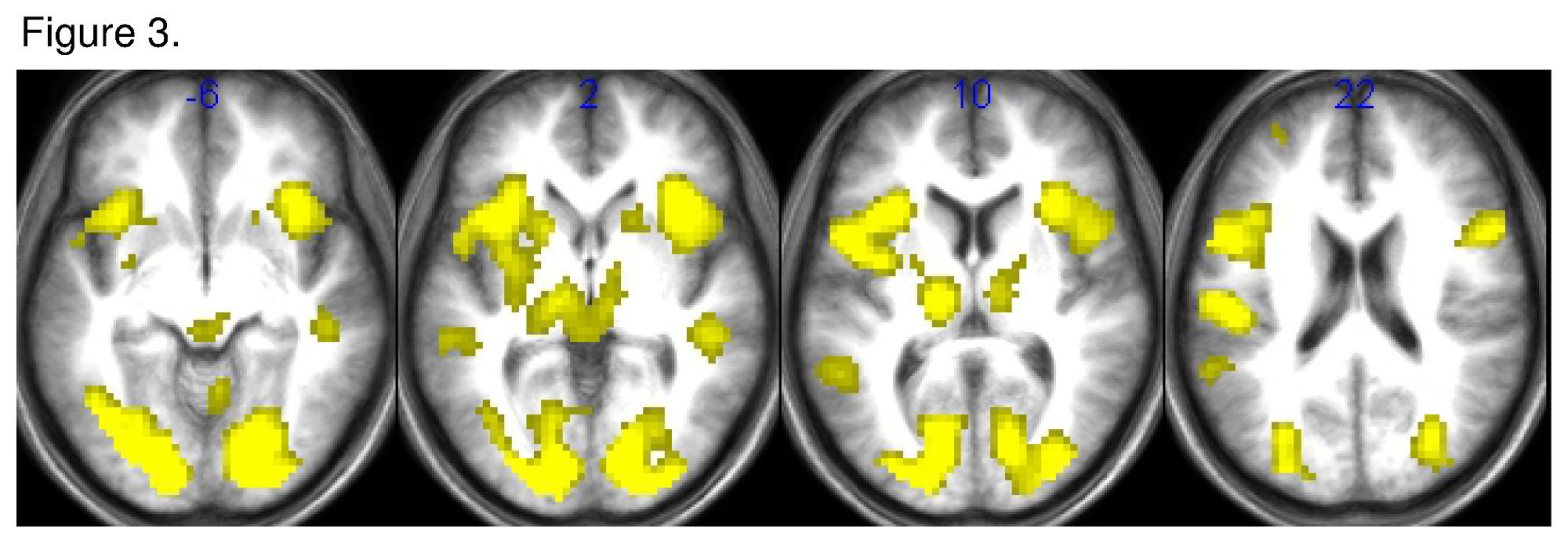
Statistical parametric maps for the contrast [ultimatum game: main effect of human opponent fair + unfair] in healthy subjects (*n* = 29). Only activations with t > 3.4 were displayed; Coordinates are given in MNI-space.

We first investigated the activation pattern associated with the UG. The contrast [UG: main effect of human opponent fair + unfair] showed a consistent set of regions associated with fair and unfair offers, e.g. the right ventral striatum, the bilateral anterior insula, the bilateral DLPFC, and the retrosplenial cortex (Table 1; Figure 3). The contrast ‘UG: positive interaction opponent x performance’ revealed significant activations in the right (MNI: 15, 11, 1) and left ventral striatum (MNI: -15, 8, 4), the right (MNI: 9, 53, 16) and left (MNI: -6, 62, 13) ventromedial prefrontal cortex (VMPFC), and the right insula (Table 1). All activations survived a voxel-wise family-wise error correction with p < 0.05 for an extent k > 10 voxel after Small Volume Correction.

**Table 1:** Activations in response to the ultimatum game.

	Region		Coordinates [MNI]	t-value	z-value
contrast: [ultimatum game: ‘positive interaction opponent x performance’] for *n* = 29					
	right ventral striatum^1^		15, 11, 1	4.30	4.12
	left ventral striatum^1^		-15, 8, 4	3.4	3.3
	right VMPFC^1^		9, 53, 13	3.82	3.69
	left VMPFC^1^		-6, 62, 13	3.6	3.49
	right insula^1^		42, 17, -8	3.09	3.02
contrast: [ultimatum game: main effect of human opponent fair + unfair] for *n* = 29					
	right ventral striatum^2^		18, 17, -2	4.05	3.90
	left dorsomedial thalamus		-15, -19, 10	7.24	6.52
	right retrosplenial cortex		3, -25, 31	5.70	5.32
	right anterior insula		39, 17, 1	8.00	7.08
	left anterior insula		-36, 17, 1	7.62	6.80
	right calcarine		15, -85, 1	12.46	> 8.0
	left calcarine		-12, -88, 4	11.66	> 8.0
	right DLPFC		54, 8, 25	6.30	5.80
	left DLPFC		-48, 5, 31	7.60	6.79
	left supplementary motor area^3^		-3, 5, 58	10.64	> 8.0
	left postcentral gyrus (BA40)		-48, -34, 49	11.92	> 8.0
	left precentral gyrus (BA6)		-45, -1, 46	7.57	6.76
contrast [ultimatum game: ‘positive effect of all conditions’] for *n* = 12					
	right DLPFC^4^		54, 8, 28	6.26	5.26
	left DLPFC^4^		-54, 8, 28	7.80	6.15

^1^initial threshold *p*[uncorr.] < 0.001, *k* > 10. All activations survived FWE correction (*p* < 0.05) on voxel-level after Small Volume Correction (S.V.C.; 5 mm radius).

^2^initial threshold *p*[uncorr.] < 0.001, *k* > 20. Only activations surviving FWE correction with p < 0.05 on voxel-level were reported. Regarding the right ventral striatum S.V.C. (radius 5 mm) was applied.

^3^extending to the right

^4^ *p*[FWE] < 0.05, *k* > 20.

*Abbreviations*: BA: Brodmann Area; DLPFC: dorsolateral prefrontal cortex; VMPFC: ventromedial prefrontal cortex.

Based on the functional localiser approach, we extracted the percent signal change for the DG conditions based on the above-mentioned regions derived from the UG.

**Figure 4:**
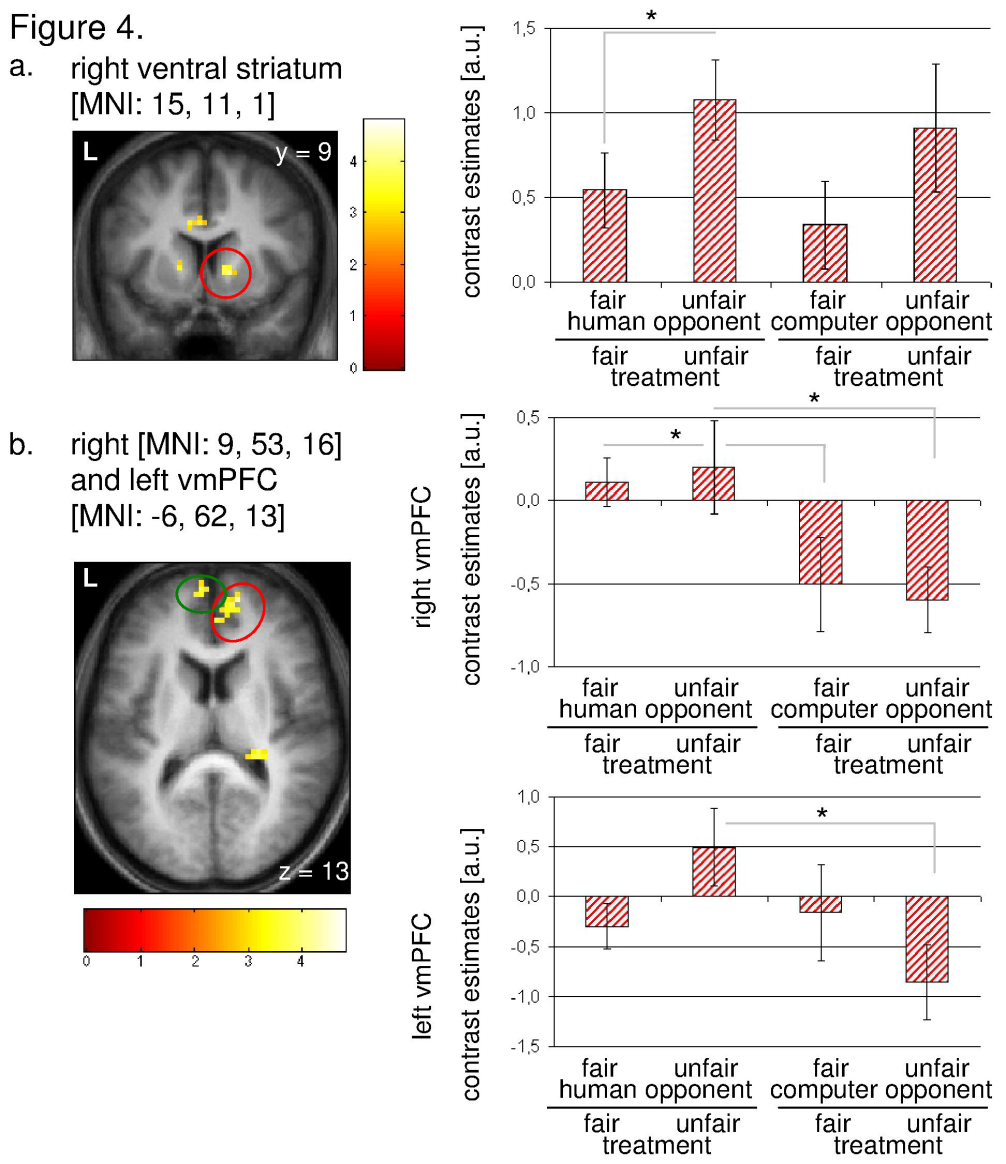
Contrast [ultimatum game: ‘positive interaction opponent x performance] and signal changes derived from the dictator game in healthy subjects (*n* = 29). a–b. Statistical parametric maps for the above mentioned contrast and percent signal change for the decision period derived from the dictator game in the (a) right ventral striatum, and (b) right and left ventromedial prefrontal cortex. The regions of interest are circled. All statistical parametric maps are thresholded at *p*[uncorr.] < 0.001 for *k* > 10. * p < 0.05. Error bar represents S.E.M.

In this context, unfair treatment of a previously unfair human player produced a significantly enhanced striatal activation compared to fair treatment of previously fair opponents (right VS: paired-t_23_ = -1.998; p_one-sided_ = 0.029). In addition, significantly enhanced activation was found in the right VMPFC between the conditions ‘fair treatment of previously fair opponent’ and ‘fair treatment of computer opponent’ (paired-t_23_ = 1.83; p_one-sided_ = 0.04), and between ‘unfair treatment of previously unfair opponent’ and ‘unfair treatment of computer opponent’ (paired-t_23_ = 2.043; p_one-sided_ = 0.026), respectively. In addition, a significant differentiation between the condition ‘unfair treatment of previously unfair opponent’ and ‘unfair treatment of computer opponent’ (paired-t_23_ = 2.191; p_one-sided_ = 0.019) was observed in the left VMPFC (Figure 4). Regarding the right insula and the left ventral striatum only non-significant results were obtained. Individual subject data for the DG is presented in Table S1.

**Figure 5:**
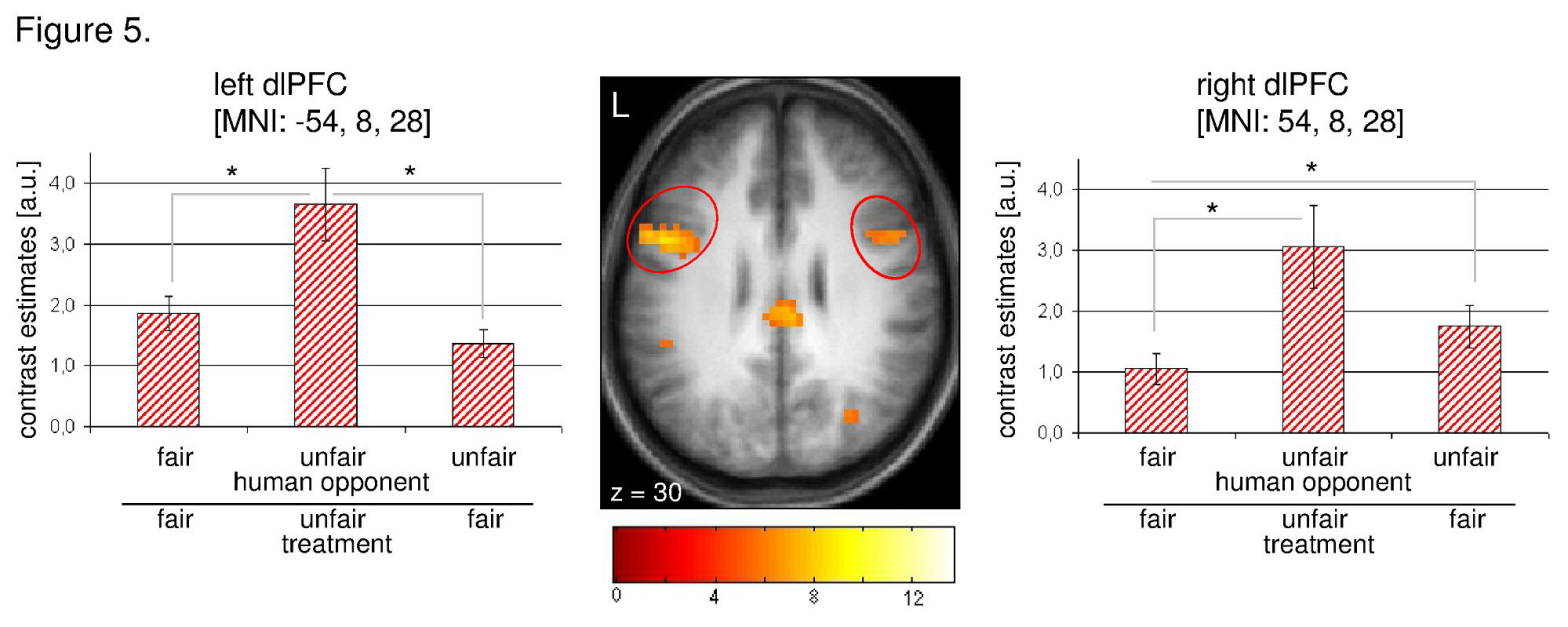
Contrast [ultimatum game: ‘positive effect of all conditions’] and signal changes derived from the dictator game in healthy subjects **(*n* = 12).** Statistical parametric maps for the above mentioned contrast and percent signal change for the decision period derived from the dictator game in the (a) right dorsolateral prefrontal cortex (MNI: 54, 8, 28), and (b) left dorsolateral prefrontal cortex (MNI: -54, 8, 28). The regions of interest are circled in red. All statistical parametric maps are thresholded at *p*[FWE] < 0.05 for *k* > 20. ● p < 0.05. Error bar represents S.E.M.

A subgroup of our study population (12/20) showed a tendency to treat previously unfair opponents in fair ways. Here, we concentrated our analysis on the right (MNI: 54, 8, 28) and left (MNI: -54, 8, 28) dorsolateral prefrontal cortex (DLPFC), because previous work has suggested that these regions are involved in controlling prepotent emotional responses. We detected a significant difference between the conditions ‘fair treatment of previously fair opponent’ and ‘unfair treatment of previously unfair opponent’ (paired-t_11_ = -2.653; p_one-sided_ = 0.011) in the right DLPFC, whereby the activation in the latter condition was greater than in the former. Notably, subjects showed a significantly enhanced activation of the right DLPFC concerning the condition ‘fair treatment of previously unfair opponent’ compared to ‘fair treatment of previously fair opponent’ (paired-t_11_ = 1.853; p_one-sided_ = 0.045). In the left DLPFC we observed an enhanced neuronal response regarding the condition ‘unfair treatment of previously unfair opponent’ compared to ‘fair treatment of previously fair opponent’ (paired-t_11_ = 1.979; p_one-sided_ = 0.037), and compared to ‘fair treatment of previously unfair opponent’ (paired-t_11_ = 2.583; p_one-sided_ = 0.013) (Figure 5).

## Discussion

Research has shown that humans have evolved psychological mechanisms to maintain cooperation between genetically unrelated individuals [1]. Social systems involving exchange based on reciprocality critically depend on the ability of individuals to discover others’ non-cooperative behaviour or defection. In addition, the prevention of defection entails the aversion of inequity, the implementation of fairness norms, and, once a violation of social rules has occurred, measures of reducing the likelihood of future defection. In interpersonal relationships, people respond to defection by unconsciously employing a calculus of the risk of future harm. This helps to guide one’s decision to either choose a retaliatory or a reconciliatory strategy, depending on the costs that the respective choice incurs [6].

In contrast to previous neuroimaging research in this domain, we were specifically interested in the question whether or not individuals would retaliate unfair behaviour and reciprocate fair behaviour when not explicitly instructed to do so. Accordingly, we designed a two-step paradigm in which subjects first acted as recipients in a UG, and subsequently as a proposer in a DG, in which the (virtual) proposers in the UG now assumed the role of a (passive) recipient. We predicted that individuals would easily recognise and distinguish fair from unfair proponents in the UG, and by and large, respond in a tit-for-tat fashion; that is, unfair behaviour experienced in the UG would elicit unfair offers in the DG to the previously unfair opponents in the UG, whereas fair behaviour in the UG would be reciprocated in the DG. In addition, we expected that a small fraction of subjects would abstain from punishing previously unfair opponents, but rather, “forgive” their unfairness by treating them in fair ways.

In accordance with predictions, a graded rejection response was found in the UG, with rejection rates rising according to the degree of unfairness of the offer. This behavioural pattern was associated with bilateral anterior insula activation as well as activation of the right ventral striatum, the bilateral DLPFC, and the retrosplenial cortex, which is in line with other neuroimaging studies [10]. In the DG, where roles were reversed, the majority of unfair proponents in the UG were treated unfairly, whereas fair proponents were treated fairly. An enhanced neuronal response of the right ventral striatum was observed when subjects treated previously unfair opponents unfairly. This finding is compatible with previous work showing that retribution is associated with striatal activity, suggesting a pleasurable effect of taking “revenge” [10], and similar striatal activation has been reported in experimental conditions where subjects experienced the emotion of “schadenfreude” [32,33]. Surprisingly, and somewhat at odds with the debriefing of the participants indicating that they comprehended the difference between human and computer condition, the behavioural responses in the UG were similar in the human, as compared to the computer condition. Interestingly, however, the neural signature associated with decision-making indicates that subjects clearly distinguished between human and computer condition. This was revealed by significantly enhanced activation in the right VMPFC when subjects responded to fair and unfair behaviour of human opponents as compared to the respective computer conditions. The VMPFC is a region that is known to be specifically involved in mentalising or “theory of mind” [34], suggesting that the attribution of mental states only occurred in the human condition.

Our findings are highly interesting with regard to recent accounts of the evolved function of revenge. According to McCullough et al. [6], revenge is a “response to harm imposition or a benefit withholding that was caused by a mechanism to deter cost-imposition or benefit-withholdings in the future”. Benefit withholding is exactly what happened in the UG that preceded the DG, suggesting that deterring someone from continuous benefit withholding was involved in the decision making in the DG. The presence of striatal activity during the retaliatory act further supports the view that experiencing pleasure through retribution is probably part of the biological underpinnings that were favoured by selection [6].

Equally interestingly was the observation that an unexpectedly high number of individuals refrained from retaliatory punishment. Instead, they treated previously unfair proposers in the DG fairly. Put another way, they inhibited their tendency to rebuff unfairness and instead, signalled their willingness to return to constructive relations. This “forgiveness” behaviour was accompanied by activation of the right DLPFC, whereas we did not observe striatal activation in this condition. This finding warrants a more detailed discussion, since the role of the DLPFC in economic decision-making has instilled debate over the question whether human behaviour is guided largely by selfish motives or altruism and empathy [35,36]. Studies using brain stimulation techniques such as repetitive transcranial magnetic stimulation (rTMS) have been particularly informative in this regard. rTMS is a tool that allows examining the causal role of cortical areas in task performance by producing transient “virtual lesions” on the cortex surface [37]. In one such experiment the inhibition of the right DLPFC led to a greater acceptance rate of unfair offers in an UG, compared to sham stimulation [38]. Moreover, in the sham condition, unfair offers were rejected faster than they were accepted and this effect was reversed after inhibition of the right DLPFC, suggesting that the rejection of unfairness was the “default” reaction that was controlled by the right DLPFC. Similarly, Knoch and colleagues [19] found that subjects rejected unfair offers less often after inhibition of the right DLPFC (but not after rTMS to the left DLPFC or sham stimulation). However, while subjects were affected in their fairness-related behaviour, their fairness judgement was unchanged, which suggests that, while subjects were well able to recognize the proposers’ unfairness, they were apparently unable to resist selfish motives of resource maximization, suggesting that the function of the right DLPFC is to implement culturally acquired fairness norms by resisting selfish motives [19]. To unravel these opposing interpretations of similar findings, we recently studied the effect of rTMS on costly punishment in a DG with the option to punish observed unfairness in the role of a third party. Here, the inhibition of the right, but not the left, DLPFC increased costly punishment. In particular, individual differences in empathy moderated the rTMS effect in a direction suggesting that the rTMS effect was stronger in individuals with lower empathy scores compared to individuals with higher empathy scores. Accordingly, we tentatively interpreted this result to suggest that costly punishment is not necessarily based on empathetic concern for others, but perhaps linked to a prepotent emotional response to avoid inequity, which can be overridden by cognitive control mechanisms involving the right DLPFC [39]. With regard to the present neuroimaging study, our findings concerning “forgiveness” could theoretically be interpreted in either way, that is, the activation of the DLPFC may indicate the overriding of an emotional response to reject unfairness, or the implementation of culturally acquired moral rules, perhaps in the form of social desirability.

A recent functional brain imaging study showed differential activation of both right and left DLPFC during revenge-like behaviour and third-party punishment. Specifically, the left DLPFC was less activated when individuals chose to retaliate (compared to no punishment), but more active when participants chose to engage in third-party punishment, whereas the right DLPFC was activated during trials involving weak third-party punishment [40]. This finding is at odds with our results showing that both right and left DLPFC were active during both retaliation and “forgiveness” behaviour (and even more strongly during retaliation as compared to “forgiveness”), whereas only the right DLPFC differentiated between fair treatment of fair opponents and fair treatment of unfair opponents (i.e. “forgiveness”). Along similar lines to our work, Buckholtz et al. [41] reported that when participants were asked to assess the responsibility and impose punishment to (virtual) perpetrators of social rules during fMRI, the right DLPFC was active during the evaluation of the responsibility of norm violators, suggesting that this brain region has a key role in the decision whether and when to punish or not to punish [41]. Likewise, a recent combined rTMS-fMRI using a UG revealed that the connectivity between the DLPFC and the VMPFC was disrupted by right but not left rTMS. This apparently led to an impaired evaluation of the fairness of offers, such that normative decision-making was impaired, indicated by a higher acceptance rate of unfair offers after right, compared to left rTMS [42]. Again, this underscores the hypothesis that the right DLPFC may also be more involved in the decision of whether or not to retaliate or “forgive” previously experienced unfairness.

The present study has some limitations. One is that, in contrast to the UGs used in previous neuroimaging studies, we applied a modified version with only four characters acting as proposers. Subjects were instructed that all human opponents represented real human characters and that the subjects’ decision in the UG and the DG would influence the monetary gain of their opponents, as well as their own payment. For this reason, all human opponents acted in more or less predictable ways, which made a direct comparison between fair and unfair offers difficult. Moreover, with regard to the analysis of “forgiveness” behaviour, the number of subjects was relatively small, suggesting that the findings should be interpreted with caution.

In summary, the present study confirms previous findings suggesting that people, when responding to violations of fairness rules in a tit-for-tat fashion, activate a neural network that in previous studies has been linked to the evaluation of one’s economic pay-off and others’ transgressions of rules of social exchange. Behaviourally, individuals tended to retaliate previously experienced unfairness and to return cooperation. A significant proportion of subjects, however, were willing to not punish unfairness. The motivation of this behaviour is not entirely clear; it could simply be that these individuals considered it unnecessary to deter rule violators from future benefit withholding. In our view, a more parsimonious explanation is that subjects evaluated the potential benefit of re-installing cooperation, indicated by the activation of the DLPFC, which could be consistent with its potential role in suppressing prepotent emotional responses to unfairness [36,37]. However, the observation that the DLPFC was activated even more strongly during retaliation suggests that this region may contribute more generally to the decision whether or not to deter from future defection or engage in reparative altruism. Future studies also ought to take into account individual differences in empathetic concern for others and cooperation.

## Supporting Information

Table S1 **Individual activation scores in the Dictator Game condition in arbitrary units [a.u.].**
(DOC)
