# Supplementary material for: “An Eye for an Eye”? Neural Correlates of Retribution and Forgiveness
Source: PLoS One. 2013 Aug 29;8(8):e73519. doi: 10.1371/journal.pone.0073519 (PMC3756996; doi:10.1371/journal.pone.0073519)
Supplement: Table S1 — Individual activation scores in the Dictator Game condition in arbitrary units [a.u.]. (DOC) [file pone.0073519.s001.doc]

Table S1. Individual activation scores in the Dictator Game condition in arbitrary units [a.u.]

| Brain region | right ventral striatum | MNI [15, 11, 1] |  |  |
| --- | --- | --- | --- | --- |
| Condition | fair-fair | unfair-unfair | comp-fair | comp-unfair |
| S01 | 1.200 | 3.418 | -0.291 | 0.566 |
| S02 | 2.484 | 0.711 | 0.416 | 2.281 |
| S03 | -0.482 | -0.122 | -0.301 | -0.956 |
| S04 | -0.206 | 1.960 | 1.026 | 0.540 |
| S05 | 0.956 | 0.294 | 1.912 | -0.345 |
| S06 | 0.499 | 2.065 | -0.424 | 0.862 |
| S07 | 0.621 | 0.319 | -0.587 | 0.902 |
| S08 | 0.040 | 0.416 | -0.373 | -2.006 |
| S09 | -1.720 | -0.356 | 0.278 | 2.070 |
| S10 | 0.418 | 1.792 | 0.653 | 0.839 |
| S11 | 0.995 | 0.797 | 0.228 | -2.335 |
| S12 | 0.949 | 0.665 | -0.742 | 1.904 |
| S13 | 0.739 | 1.325 | 0.689 | -0.746 |
| S14 | -0.138 | 0.045 | 2.889 | -0.465 |
| S15 | 0.431 | 1.150 | 1.325 | 0.214 |
| S16 | 1.764 | 0.272 | 2.230 | 0.566 |
| S17 | 0.769 | 3.144 | -0.644 | 6.574 |
| S18 | 1.945 | 0.652 | 1.370 | 1.697 |
| S19 | 0.245 | -0.890 | -0.713 | 1.459 |
| S20 | -1.770 | -0.050 | -1.797 | 4.068 |
| S21 | -0.826 | 2.000 | -1.596 | 1.256 |
| S22 | 0.442 | 2.116 | 2.923 | 1.840 |
| S23 | 1.374 | 0.895 | 0.068 | 0.314 |
| S24 | 2.321 | 3.270 | -0.424 | 0.724 |
|  |  |  |  |  |
| mean | 0.544 | 1.079 | 0.338 | 0.909 |
| SD | 1.082 | 1.168 | 1.267 | 1.843 |
| mean + 3SD | 3.791 | 4.583 | 4.140 | 6.439 |
| mean - 3SD | -2.703 | -2.426 | -3.464 | -4.621 |
|  |  |  |  |  |
| Brain region | R vmPFC | MNI [9, 53, 16] |  |  |
| Condition | fair-fair | unfair-unfair | comp-fair | comp-unfair |
| S01 | 0.386 | 2.772 | 0.089 | 0.178 |
| S02 | -0.838 | -0.765 | -2.315 | -0.887 |
| S03 | -0.179 | 0.849 | -0.788 | -1.605 |
| S04 | -0.786 | -0.501 | -0.660 | -0.982 |
| S05 | 0.238 | 0.828 | -1.264 | 0.102 |
| S06 | 0.450 | 1.101 | 0.729 | -1.004 |
| S07 | 0.020 | 0.950 | 0.323 | -1.124 |
| S08 | -0.557 | 0.545 | -1.629 | -0.312 |
| S09 | -0.024 | -0.110 | -0.020 | -0.872 |
| S10 | 0.023 | 0.180 | 0.416 | 0.604 |
| S11 | 1.123 | -0.366 | -4.388 | -0.224 |
| S12 | -0.358 | 0.662 | 0.476 | -1.474 |
| S13 | 0.420 | -0.214 | 0.258 | 0.625 |
| S14 | -0.999 | -0.555 | 0.667 | 1.174 |
| S15 | -0.640 | -0.439 | -0.548 | -1.484 |
| S16 | -0.015 | 0.135 | 0.473 | 0.874 |
| S17 | 0.129 | 3.677 | -2.213 | -2.151 |
| S18 | -0.222 | -0.942 | 1.734 | 0.603 |
| S19 | 0.593 | -3.308 | -2.966 | -0.354 |
| S20 | -0.997 | 0.065 | -0.307 | -1.285 |
| S21 | 1.433 | 1.466 | -0.971 | -1.635 |
| S22 | 1.218 | -1.510 | -0.060 | 0.186 |
| S23 | 1.265 | -0.361 | 0.843 | -2.044 |
| S24 | 0.979 | 0.649 | 0.032 | -1.280 |
|  |  |  |  |  |
| mean | 0.111 | 0.200 | -0.504 | -0.599 |
| SD | 0.730 | 1.366 | 1.389 | 0.967 |
| mean + 3SD | 2.301 | 4.297 | 3.663 | 2.302 |
| mean - 3SD | -2.079 | -3.896 | -4.670 | -3.500 |
|  |  |  |  |  |
| Brain region | L vmPFC | MNI [-6, 62, 13] |  |  |
| Condition | fair-fair | unfair-unfair | comp-fair | comp-unfair |
| S01 | 1.069 | 0.554 | 0.825 | 0.328 |
| S02 | -1.047 | -0.588 | 2.464 | -1.107 |
| S03 | -1.168 | 0.311 | -1.232 | -3.229 |
| S04 | -0.828 | -0.414 | -0.308 | -2.061 |
| S05 | 0.323 | 2.717 | -1.860 | 0.684 |
| S06 | -0.683 | 0.275 | 0.712 | -0.192 |
| S07 | 0.073 | 1.847 | -0.259 | -2.441 |
| S08 | -1.612 | 0.754 | -2.146 | -0.968 |
| S09 | -0.478 | -0.159 | 0.067 | -0.717 |
| S10 | 0.367 | -0.273 | 0.134 | 0.443 |
| S11 | 1.028 | -0.467 | -6.940 | -0.846 |
| S12 | -0.472 | 0.597 | -0.801 | -2.375 |
| S13 | -0.878 | 0.453 | -0.285 | 0.481 |
| S14 | -1.163 | -1.059 | 0.512 | -1.495 |
| S15 | -0.823 | -0.706 | -2.084 | -3.001 |
| S16 | 0.307 | 0.701 | 0.455 | 0.880 |
| S17 | -0.445 | 6.690 | 5.858 | -3.422 |
| S18 | -0.101 | -1.047 | 1.497 | -0.575 |
| S19 | 1.850 | -2.670 | -2.167 | 4.862 |
| S20 | -2.322 | 0.033 | -0.898 | -1.239 |
| S21 | -1.925 | 3.932 | -1.539 | -1.823 |
| S22 | 2.294 | 0.395 | 3.760 | -1.089 |
| S23 | -0.717 | -1.773 | -0.475 | -3.163 |
| S24 | 0.193 | 1.803 | 0.916 | 1.490 |
|  |  |  |  |  |
| mean | -0.298 | 0.496 | -0.158 | -0.857 |
| SD | 1.111 | 1.916 | 2.375 | 1.854 |
| mean + 3SD | 3.036 | 6.244 | 6.967 | 4.704 |
| mean - 3SD | -3.632 | -5.252 | -7.284 | -6.418 |

Legend: L = left; MNI = Montreal Neurological Institute; R = right; S = subject; SD = standard deviation; vmPFC = ventromedial prefrontal cortex.
